# Absence of cyclin D2 expression is associated with promoter hypermethylation in gastric cancer

**DOI:** 10.1038/sj.bjc.6600940

**Published:** 2003-05-13

**Authors:** J Yu, W K Leung, M P A Ebert, R W L Leong, P C H Tse, M W Y Chan, A H C Bai, K F To, P Malfertheiner, J J Y Sung

**Affiliations:** 1 Department of Medicine & Therapeutics, Prince of Wales Hospital, The Chinese University of Hong Kong, Shatin, Hong Kong, China; 2 Department of Gastroenterology, Hepatology, and Infectious Diseases, Otto-von-Guericke University, Magdeburg, Germany; 3 Department of Anatomical & Cellular Pathology, Prince of Wales Hospital, The Chinese University of Hong Kong, Shatin, Hong Kong, China

**Keywords:** Q1cyclin D2, gastric carcinoma, hypermethylation, demethylation

## Abstract

Expression of cyclin D2 is absent in 30–70% of gastric cancers. We investigated the role of promoter hypermethylation in the transcriptional silencing of *cyclin D2* in five gastric cell lines and 47 primary gastric carcinomas. CpG island methylation status of the *cyclin D2* gene was studied by methylation-specific polymerase chain reaction and bisulphite sequencing. RNA and protein expression was analysed by reverse transcription–PCR and Western blot, respectively. Dense methylation of *cyclin D2* was detected in three cell lines (KATOIII, AGS and NCI-N87), which also lacked cyclin D2 mRNA and protein expression. Bisulphite DNA sequencing revealed that loss of cyclin D2 expression was closely associated with the density of methylation in the promoter region. Treatment with DNA methyltransferase inhibitor, 5-aza-2′-deoxycytidine, restored the cyclin D2 expression level in methylated gastric cells. Among the 47 primary gastric cancers, *cyclin D2* hypermethylation was detected in 23 (48.9%) cases. None of the 23 normal gastric biopsies from noncancer patients showed hypermethylation. Hypermethylation was associated with loss of mRNA (*P*<0.001) and protein (*P*=0.006) expressions. Our study showed that *cyclin D2* hypermethylation is associated with loss of cyclin D2 expression in a subset of gastric cancers, which may suggest an alternative gastric carcinogenesis pathway in the absence of cyclin D2 expression.

Cyclin D2 is involved in the regulation of the cell cycle at the point of transition from G1 to DNA synthesis. In addition to its role in cell cycle regulation, cyclin D2 is also implicated in cellular differentiation and malignant transformation (Evron *et al*, 2001). Overexpression of cyclin D2 has been reported in gastric cancer and is shown to correlate with disease progression and poor prognosis (Takano *et al*, 1999; Yu *et al*, 2001). On the other hand, cyclin D2 expression is not universal in gastric cancer. We and others have demonstrated that cyclin D2 mRNA and/or protein are absent in 30–70% of gastric cancers (Yasogawa *et al*, 1998; Takano *et al*, 1999,^2000^). These results indicate that a subset of gastric cancers arise from cyclin D2 independent pathway.

Recently, there is a growing body of evidence to suggest that promoter hypermethylation is a major mechanism for the silencingQ2 of tumour-suppressor genes (Jones and Laird, 1999; Baylin and Herman, 2000). Cytosine methylation of CpG islands located within the promoter region is generally associated with delayed replication, condensed chromatin and inhibition of transcription initiation (Baylin *et al*, 1998; Delgado *et al*, 1998; Jones and Laird, 1999). Studies including ours indicated that aberrant hypermethylation of 5′ CpG islands is one of the crucial mechanisms in the transcriptional silencing of multiple tumour-related genes in gastric cancer (Lee *et al*, 1997; Iida *et al*, 2000; Kang *et al*, 2000; Shin *et al*, 2000; Song *et al*, 2000; Leung *et al*, 2001). Recent studies suggested that cyclin D2 expression is inhibited by the aberrant methylation of the promoter region of the *cyclin D2* gene in breast cancers as well as in Epstein–Barr virus-positive Burkitt's lymphoma (Sinclair *et al*, 1995; Evron *et al*, 2001; Lehmann *et al*, 2002). However, there is no available information on the methylation status of the *cyclin D2* gene in gastric cancer. The aim of the current study was to examine the methylation status of the CpG sites in the *cyclin D2* promoter region of gastric cancer by methylation-specific PCR (MSP) and bisulphite DNA sequencing. We also correlated the expression of cyclin D2 with *cyclin D2* promoter hypermethylation in gastric cancer.

## MATERIALS AND METHODS

### Cancer cell lines and tissues

The human gastric cancer cell lines KATO III, MKN45, MKN28, AGS and NCI-N87 (N87) were obtained from Riken Cell Bank (Tsukuba, Japan) or American Type Culture Collection (ATCC; Rockville, MD, USA). All cell lines, except AGS, were maintained in RPMI medium (Gibco BRL, Rockville, MD, USA) with 10% foetal bovine serum (Gibco BRL). AGS cell line was kept in F-12K medium (ATCC) with 10% foetal bovine serum.

Gastric cancers were obtained from 47 gastrectomy patients at the time of surgery. There were 30 males and 17 females with a mean age of 64.8 years (range 36–83). In addition, normal endoscopic gastric biopsies from 23 noncancer subjects (mean age 53.3 years, range 35–77 years) were used as control. The samples were immediately snap frozen in liquid nitrogen and stored at −80°C. The remaining tissue specimens were fixed in 10% formalin and embedded in paraffin for routine histological examination and immunohistochemical analysis. All patients gave informed consent for obtaining the study specimens, and the study protocol was approved by the ethics committee of the local hospitals.

### Reverse transcription–polymerase chain reaction (RT–PCR)

**Figure 1:**
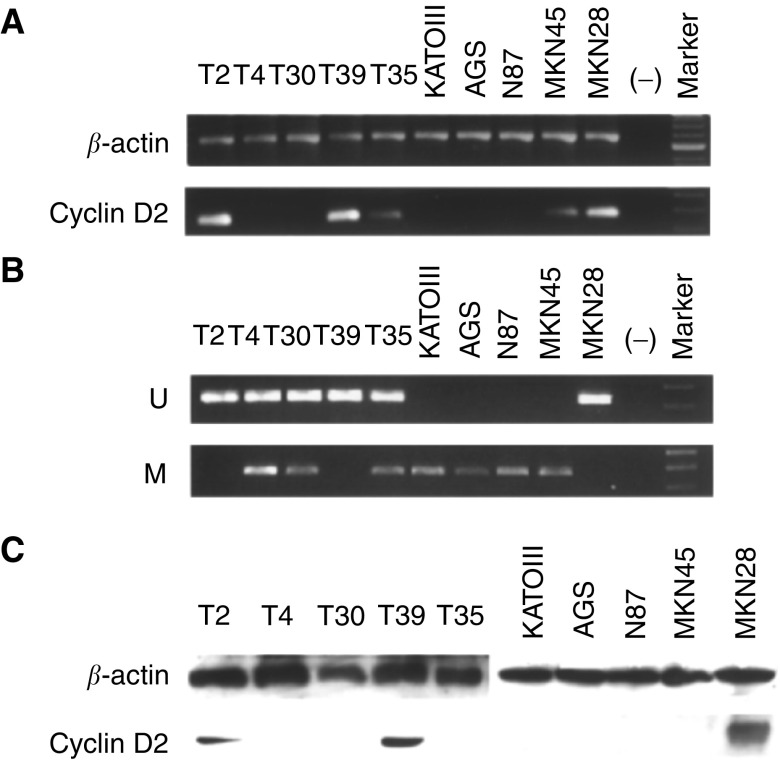
(**A**) Expression of cyclin D2 mRNA in a panel of five gastric cancer cell lines (KATO III, AGS, N87, MKN45 and MKN28) and five gastric cancer tissues (T2, T4, T30, T39 and T45) by RT–PCR. A blank control (H_2_O) was included in each PCR experiment (−). mRNA for *β*-actin was used as control for the integrity of RNA samples. A 100-bp marker was run in parallel on an agarose gel. (**B**) Methylation-specific PCR was performed after bisulphite modification of DNA. U indicates unmethylated cyclin D2 PCR products. M indicates methylated cyclin D2 PCR products. Three representative samples of methylation-positive (T4, T30 and T35) and two methylation-negative (T2 and T39) tumours are shown. DNA template-negative control (H_2_O) was also included (−). (**C**) Representative examples of Western blot analysis of cyclin D2 expression. Lysates (20 *μ*g of protein) from gastric cancer cell lines and primary gastric tumours were immunoblotted with a cyclin D2 antibody.

Gastric tissue specimens were homogenised with an ultrasound homogeniser. Total RNA was extracted by using RNA Tri Reagents (CINNA/MRC, Cincinnati, OH, USA) according to the manufacturer's protocol. Total RNA (1 *μ*g) was reverse transcribed into cDNA by using dNTP (1 mM), 5 × reverse transcription buffer (500 mM Tris-HCl pH 8.3, 250 mM KCl, 50 mM MgCl_2_ and 50 mM DTT), 16 U RNasin and 2.5 U of AMV reverse transcriptase (Gibco BRL). For PCR, the primer sequences were as follows: *cyclin D2* (Evron *et al*, 2001), (sense) 5′-CATGGAGCTGCTGTGCCACG-3′ and (antisense) 5′-CCGACCTACCTCCAGCATCC-3′; and *β-actin*, (forward) 5′-TGACGGGGTCACCCACACTGTGCCCATCTA-3′, (reverse) 5′-CTAGAAGCATTTGCGGTGGACGATGGAGGG-3′. The reaction was performed at 95°C for 1.5 min, and was followed by 35 cycles of denaturating at 95°C for 24 s, annealing at 58°C for 48 s and extension at 72°C for 1 min. The PCR products were separated on 1.5% agarose gel and saved as digital images (Uvigrab; UVItec, Cambridge, UK) (Figure 1A

).

### Western blot analysis

Proteins were extracted from cell pellets using Trizol Reagents (Gibco BRL). Protein concentrations were measured by the method of Bradford (Bio-Rad, Hercules, USA). In all, 20 *μ*g of protein was loaded per lane, separated by 10% SDS–polyacrylamide gel electrophoresis under reducing conditions, and transferred onto equilibrated polyvinylidene difluoride membrane (Amersham Biosciences Com., UK) by electroblotting. Membranes were blocked by 5% nonfat dry milk and then incubated with anticyclin D2 antibody (dilution 1 : 1000; sc-181-G, goat polyclonal antibodies; Santa Cruz Biotechnology, Santa Cruz, CA, USA) for 2.5 h at room temperature. After secondary antibody incubation, cyclin D2 was detected by the enhanced chemiluminescence detection system (Amersham Biosciences Com.) (Figure 1C).

### Immunohistochemistry

Expression of cyclin D2 protein was examined by avidin–biotin complex (ABC) immunoperoxidase method as described previously (Yu *et al*, 2001). Sections of 5 *μ*m were cut from the paraffin-embedded blocks, placed on charged glass slides, deparaffinised, rehydrated, rinsed with distilled water and washed with Tris-buffered saline (TBS). The slides were then treated with 3% hydrogen peroxide to block endogenous peroxidase activity. After blocking with 5% normal serum for 20 min, polyclonal antibody against cyclin D2 (sc181, Santa Cruz, CA, USA) was applied and incubated at 4°C overnight. After rinsing, the biotinylated secondary antibody and ABComplex/HRP (Dako A/S, Denmark) were applied. Peroxidase activity was visualised by the diaminobenzidine chromogen with 0.05% hydrogen peroxide. The sections were then counterstained with haematoxylin, dehydrated, cleared and mounted. Since this antibody may have minimal crossreaction with cyclin D1, parallel paraffin-embedded sections were used in staining for cyclin D1 immunoreactivity (Dako A/S). In most cancer cases with cyclin D2 immunoreactivity, cyclin D1 was not detected, which suggested that the staining obtained was because of cyclin D2 expression in these tumours.

### Bisulphite modification

Genomic DNA from cell lines or frozen gastric tissues was extracted by using the High Pure PCR Template Preparation kit (Roche, Germany). Genomic DNA of 1 *μ*g was treated with sodium bisulphite using the CpGenome DNA Modification Kit (Intergen, Purchase, NY, USA) according to the manufacturer's instructions. After bisulphite treatment, cytosine residues are deaminated and changed into uracil residues, but methylated cytosine remains unmodified. Differentiation between methylated and unmethylated sequences can then be made by amplification using specific primers that target either the uracil or the cytosine nucleotide.

### Methylation-specific PCR

PCR amplification was performed on the bisulphite-modified DNA samples using primer sets targeting the CpG-rich region in the *cyclin D2* promoter. The methylated and unmethylated primer sequences were based on the report by Evron and the regions of primers were numbered from the transcriptional start site (Evron *et al*, 2001): unmethylated reaction, 5′-GTTATGTTATGTTTGTTGTATG-3′ (sense, −1372 to −1350) and 5′-TAAAATCCACCAACACAATCA-3′ (antisense, −1150 to −1170), 223-bp product; and methylated reaction, 5′-TACGTGTTAGGGTCGATCG-3′ (sense, −1183 to −1165) and 5′-CGAAATATCTACGCTAAACG-3′ (antisense, −908 to −927), 276-bp product. Hot start PCR was conducted in a 25 *μ*l reaction solution containing 1 × PCR buffer, 0.25 mM each of the deoxynucleoside triphosphates, 1 mM of each primer and 1 U of *Taq* polymerase (AmpliTaq Gold; PE Applied Biosystems, Foster City, CA, USA). The temperature profile for the amplification was as follows: 12 min at 95°C, 35 cycles of denaturing at 95°C for 30 s, 45 s annealing at 52°C, 60 s extension at 72°C, and a final extension step of 5 min at 72°C. PCR products were analysed in 2% agarose gels and stained with ethidium bromide (Figure 1B). *In vitro* methylated control (positive control; Intergen) and DNA template-negative control (H_2_O) were included in each PCR. All reactions were repeated twice to ensure consistency of results.

### Bisulphite DNA sequencing

For bisulphite DNA sequencing analysis, PCR primers were designed to amplify a CpG-rich region spanning from −1220 to −883 from the transcriptional start site, which contains 27 CpG sites. Primer sequences were: 5′-TTTGTAAAGATAGTTTTGATTTAAGG-3′(−1220 to −1195 forward) and 5′-CCCCTACATCTACTAACAAAC-3′ (−883 to −903, reverse). The PCR product was cloned into the pCR4-TOPO® vector using the TOPO TA Cloning® Kit (Gibco/Invitrogen, Carlsbad, USA). Multiple clones (minimum of five) from each PCR product were sequenced using the ABI Prism Dye Terminator Cycle Sequencing Kit (PE Biosystems, Foster City, CA, USA) and the ABI Prism 310 DNA Sequencer (PE Biosystems).

### Treatment of cells with 5-aza-2′-deoxycytidine (5-azaDC)

Cells were seeded at a density of 1 × 10^6^ cells 60 mm^−1^ dish. After 24 h, cells were treated with 1, 5 or 10 *μ*M 5-azaDC (Sigma Chemical Co., USA). The same concentration of DMSO was used as a control for nonspecific solvent effect on cells. Total cellular RNA and protein were isolated 3 and 5 days after addition of 5-azaDC as described above.

### Statistical analysis

The difference between the methylated and the unmethylated groups was evaluated by *χ*^2^ test or Fisher's exact test. A two-sided *P*-value of less than 0.05 was considered statistically significant.

## RESULTS

### Methylation of *cyclin D2* is associated with transcriptional silencing in gastric cancer cell lines

By using RT–PCR and Western blotting, cyclin D2 mRNA and protein expression was found only in MKN28 but not in KATOIII, AGS and N87 cell lines (Figure 1A, C). Notably, MKN45 had reduced level of cyclin D2 mRNA expression but there was no protein expression detected. A screen for *cyclin D2* promoter methylation was performed by MSP. Hypermethylation at the CpG-rich region with no mRNA expression was detected in all three cell lines (KATOIII, AGS, N87) as well as in MKN45 (Figure 1B), but was not detected in MKN28 with strong mRNA and protein expression.

**Figure 2:**
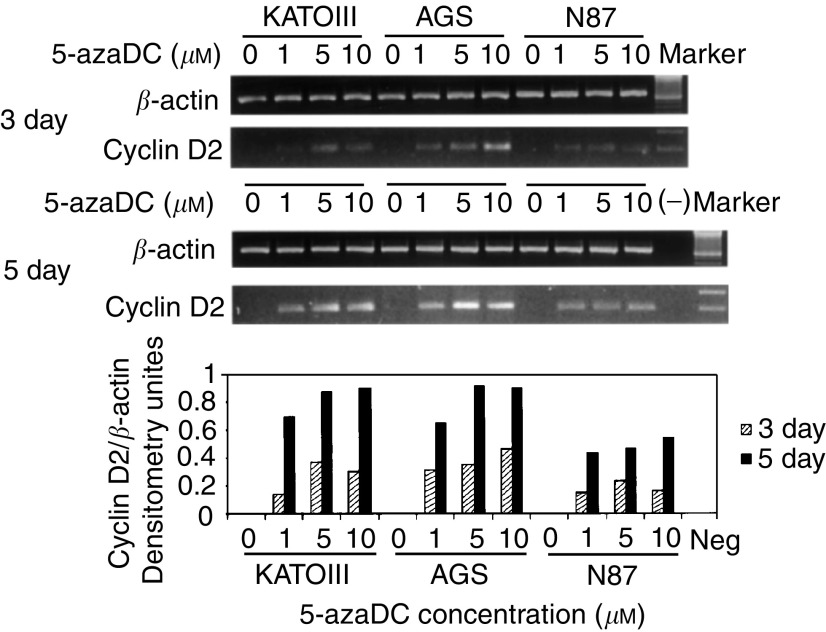
Inhibition of methylation restored cyclin D2 expression in cyclin D2-negative cell lines (KATO III, AGS and N87). Cell lines were treated with 0, 1, 5 or 10 *μ*M of 5-azaDC for 3 and 5 days, respectively. The lower figures show the levels of the cyclin D2 expression in relation to *β*-actin as measured by densitometer after treatment with different doses of 5-azaDC.

Next, we treated cyclin D2 methylated cell lines (KATOIII, AGS, N87) with the methylation inhibitor 5-azaDC (Jones, 1985). Expression of cyclin D2 was restored in all three methylated cell lines after 3 days treatment with 5-AzaDC (Figure 2

). The ability of 5-azaDC to enhance expression of cyclin D2 was more marked when cells were treated for 5 days.

### Hypermethylation leads to *cyclin D2* silencing in primary gastric tumours

Among the 47 primary gastric cancers, 23 (48.9%). had *cyclin D2* methylation detected by MSP (Figure 1B). The presence of both methylated and unmethylated bands in tumour samples reflects heterogeneity of the tumour or may represent the inclusion of normal tissues or infiltrating lymphocytes in tissue homogenates. Of the 23 methylation-positive cases, 15 (65.2%) had complete loss of cyclin D2 mRNA expression. In contrast, only three of 24 (12.5%) methylation-negative cases had lost cyclin D2 mRNA expression. There was a strong association between the lack of cyclin D2 mRNA expression and promoter hypermethylation (Table 1

**Table 1:** Association between cyclin D2 mRNA expression with promoter hypermethylation in gastric cancers

	**Cyclin D2 mRNA**		
**Cyclin D2 methylation**	**Positive**	**Negative**	**Total**
Positive	8	15	23
Negative	21	3	24
			
Total	29	18	47

*P*<0.001.

; *P*<0.001). To further demonstrate that promoter methylation of *cyclin D2* is a tumour-specific phenomenon, DNA from 23 histologically normal gastric mucosa were tested. None of these normal samples had methylation detected by MSP (data not shown).

Western blot was performed in 28 randomly selected cases of gastric cancer. In total, 10 (66.7%) of the 15 cases with promoter hypermethylation in *cyclin D2* did not express the corresponding protein. On the other hand, only two of the 13 unmethylated tumours did not express cyclin D2 protein (*P*=0.006; Table 2

**Table 2:** Association between cyclin D2 protein expression and promoter hypermethylation in human gastric cancers

	**Cyclin D2 protein**		
**Cyclin D2 methylation**	**Positive**	**Negative**	**Total**
Positive	5	10	15
Negative	11	2	13
			
Total	16	12	28

**Figure 3:**
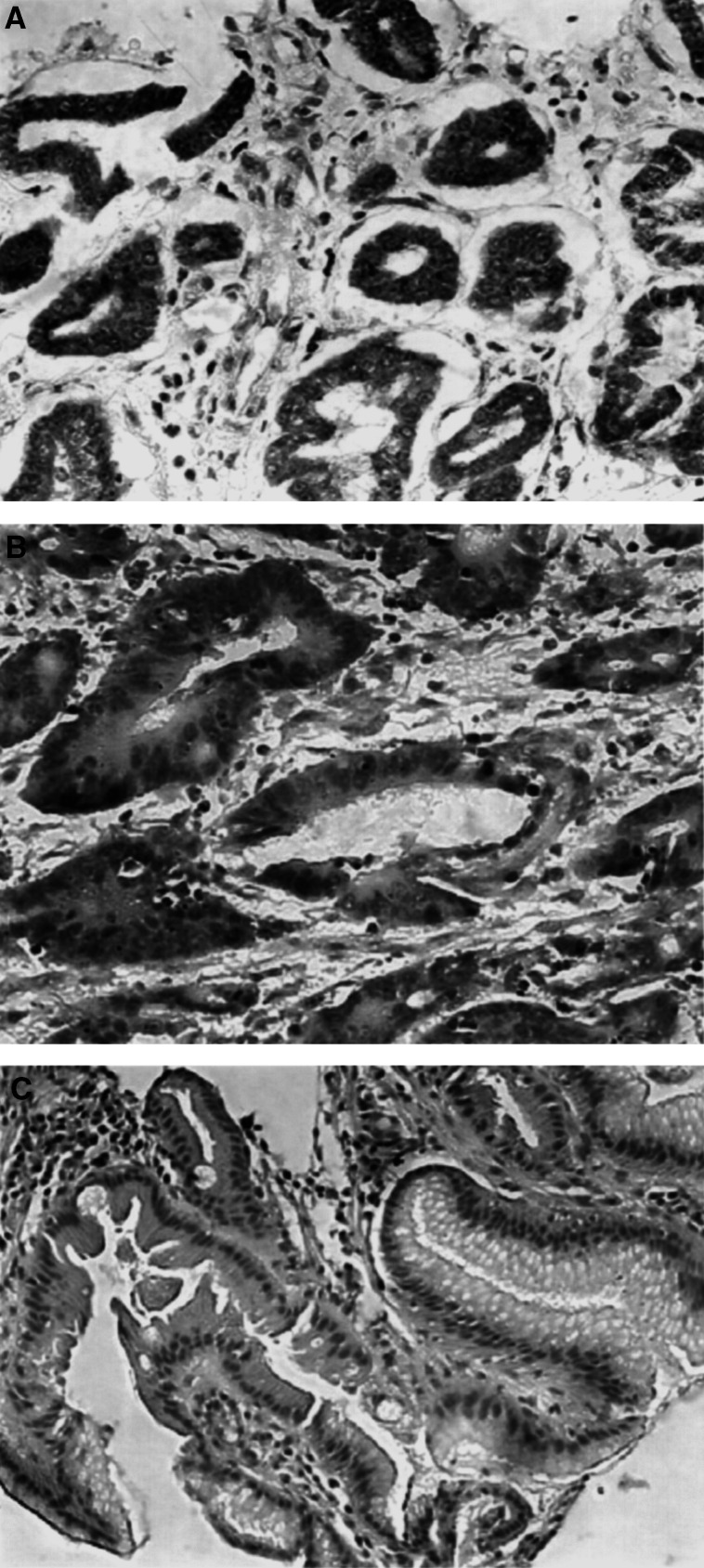
Representative immunohistochemical staining of cyclin D2. (**A**) Cyclin D2 expression was detected in gastric cancers without cyclin D2 methylation. (**B**) In gastric cancers with promoter methylation in *cyclin D2*, there was no cyclin D2 immunoreactivity detected. (**C**) Normal gastric mucosa from noncancer subjects was negative for cyclin D2.

*P*=0.006.

). In keeping with the findings by Western blot, cyclin D2 immunoreactivity was detected in the cytoplasm and nucleus of gastric cancers without methylation (Figure 3A

). In methylated tumours (Figure 3B) and in normal gastric tissues (Figure 3C), there was no cyclin D2 immunoreactivity detected.

We next examined the potential association between clinicopathological and molecular characteristics of gastric cancer with *cyclin D2* methylation. *cyclin D2* methylation was more common in cancer patients ⩾60 years of age (78.3 *vs* 35.7%, *P*=0.01; Table 3

**Table 3:** Association between cyclin D2 methylation and clinicopathological characteristics of gastric cancers

**Variable**	**Category**	**Total no.**	**Cyclin D2 methylation (%)**	***P*-value**
Sex	Male	30	17 (56.7)	0.159
	Female	17	6 (35.3)	
				
Age (years)	⩾60	23	18 (78.3)	0.01
	<60	14	5 (35.7)	
				
Lymph node metastasis	Present	32	16 (50.0)	0.831
	Absent	15	7 (46.7)	
				
Depth of tumour				0.722
	T1	4	2 (50.0)	
	T2	20	9 (45.0)	
	T3	11	7 (63.6)	
	T4	12	5 (41.7)	
				
Lauren classification	Intestinal type	16	6 (37.5)	0.429
	Diffuse type	26	13 (50.0)	

). However, methylation in *cyclin D2* was not detected in any of the 23 normal gastric biopsies including 10 patients who were ⩾60 years, suggesting that methylation is not an age-related phenomenon in normal gastric epithelium. Otherwise, there was no significant association between *cyclin D2* methylation and clinicopathological parameters of tumour including tumour classification, lymph node status and pathological grading.

### Bisulphite DNA sequencing

**Figure 4:**
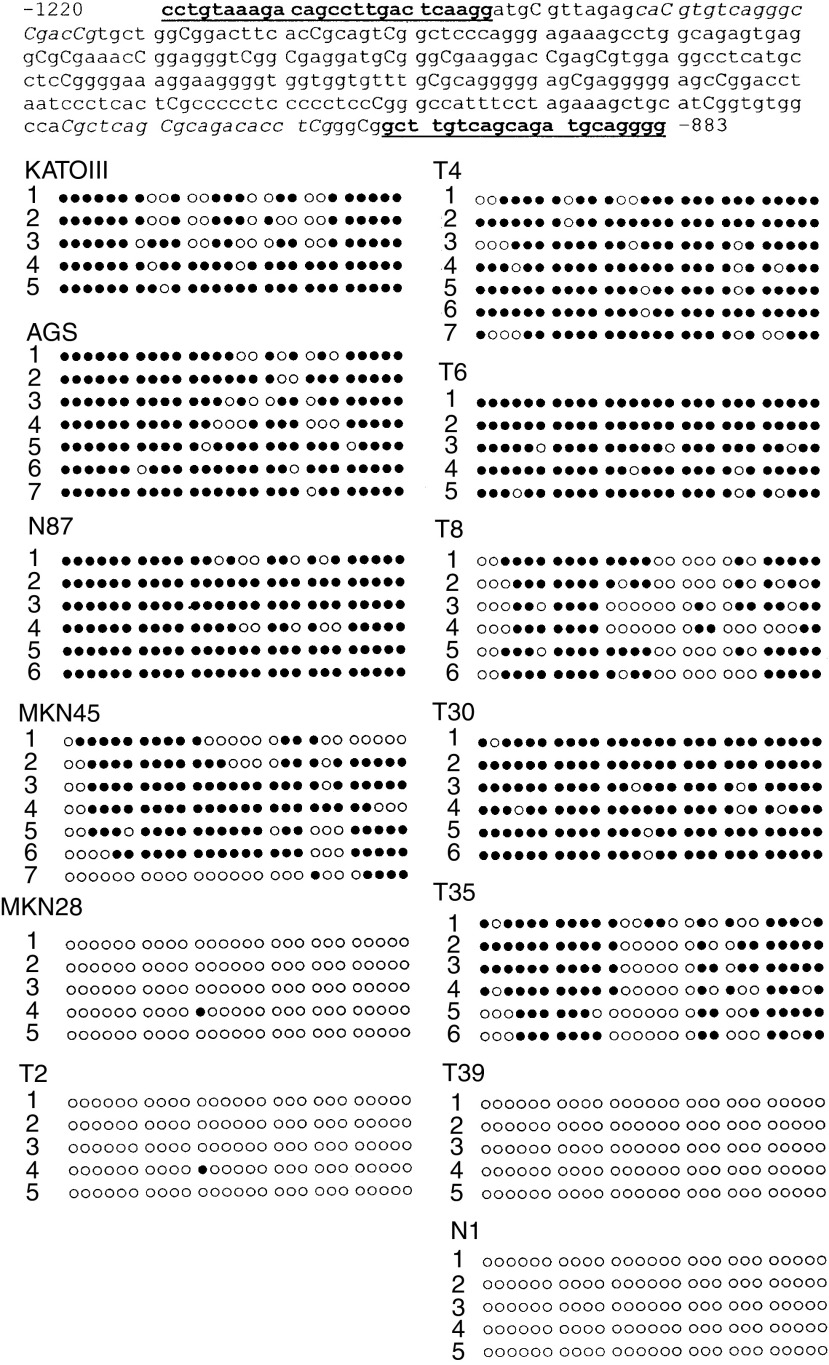
Bisulphite sequencing of *cyclin D2* promoter region. The nucleotide sequence from −1220 to −883 of the cyclin D2 gene is shown. The individual CpG sites between two PCR primers are numbered sequentially. Cytosines at the CpG site are in capitalisation. The bisulphite sequencing PCR primers are bold and underlined whereas the MSP primers are shown as italic. DNA from five gastric cell lines, two cyclin D2-positive cancers (T2, T39), two cancers with low cyclin D2 expression (T8, T39) and three cyclin D2-negative (T4, T6, T30) cancers as well as one normal gastric tissue (N1) were bisulphite-treated, PCR-amplified and subcloned. The sequencing results from five to eight clones for each cell line and samples are presented. Each horizontal line represents the sequencing result of one subclone. CpG sites within 48 bp are shown as one block. Methylated CpG sites are shown as ‘•’ whereas ‘○’ indicate unmethylated CpG sites.

To verify the MSP findings and to study the extent of promoter methylation, bisulphite DNA sequencing was performed. The CpG-rich region of the *cyclin D2* promoter between the nucleotides −1220 and −883 was sequenced after bisulphite modification (Figure 4

). Bisulphite genomic sequencing of the representative PCR products showed that all the cytosines at non-CpG sites were converted to thymine. This excluded the possibility that successful amplification was attributable to incomplete bisulphite conversion. Moreover, the results of MSP and bisulphite sequencing were concordant in both cell lines and primary gastric cancers, indicating that it is appropriate to draw inferences from the results of the MSP.

As shown in Figure 4, the CpG island exhibited extensive methylation in the three cell lines without cyclin D2 expression (KATOIII, AGS, N87). In contrast, there was no methylation in the MKN28 cell lines with positive cyclin D2 expression. Notably, the percentage of methylation ranged from 18.5 to 88.9% in the MKN45 cell line (Figure 4). This partial methylation may explain the low cyclin D2 mRNA expression in the MKN45 cell line as detected by RT–PCR.

Bisulphite sequencing was also performed in seven randomly selected gastric cancers: two with cyclin D2 expression (T2, T39), two with low cyclin D2 expression (T8, T35) and three cyclin D2-negative (T4, T6, T30) cancers (Figure 4). The three cases (T4, T6 and T30) that showed hypermethylation by MSP had densely methylated alleles by bisulphite sequencing whereas the two cases with low cyclin D2 expression (T8 and T35) had partially methylated CpG sites. In contrast, the two tumours with strong cyclin D2 expression (T2, T39) had virtually no methylation detected. In addition, bisulphite sequencing of normal gastric mucosa (N1) showed the absence of methylation in the promoter region.

## DISCUSSION

DNA methylation forms repressive chromatin (Brooks *et al*, 1996; Bird and Wolffe, 1999; Baylin and Herman, 2000) and affects gene expression (Baylin *et al*, 1998; Bird and Wolffe, 1999; Jones and Laird, 1999). Herein, we tested the association between *cyclin D2* promoter hypermethylation and loss of cyclin D2 expression in gastric cancer. We first examined the promoter methylation status and expression of cyclin D2 in gastric cancer cell lines. Three gastric cancer cell lines (KATOIII, AGS and NCI-N87) with dense methylation at the CpG islands do not express cyclin D2 mRNA and protein. Treatment with 5-azaDC induced demethylation of the CpG islands with reactivation of gene expression in these cyclin D2-negative hypermethylated cell lines. The MKN28 cell line had almost no methylation and displayed strong cyclin D2 expression. Interestingly, the MKN45 cell line was noticed to have a reduced level of cyclin mRNA expression, which may be related to the partial methylation of the promoter region. The same observation was also found in primary human gastric cancers in which the density of methylation appears to have an inverse association with the expression of cyclin D2. To our knowledge, this is the first comprehensive examination of the promoter region of *cyclin D2*. In keeping with our finding, Lehmann *et al* (2002) reported that there was a significant increase in quantitative changes in the methylation level from intraductal to invasive breast cancer.

Additionally, we showed that most human gastric cancers (15 out of 23, 65.2%) with *cyclin D2* promoter hypermethylation had no cyclin D2 mRNA expression whereas the majority (21 out of 24, 87.5%) of tumours with unmethylated *cyclin D2* promoter region had cyclin D2 expression. Similar results were demonstrated in protein level by Western blotting (Table 2). Taken together, these results suggest that promoter hypermethylation is a major mechanism underlying the loss of cyclin D2 function in both gastric cell lines as well as in primary gastric cancer. Moreover, it offers an explanation for the lack of cyclin D2 expression in a subset of gastric cancer (Yasogawa *et al*, 1998; Takano *et al*, 1999,^2000^). Intuitively, cyclin D2 expression may not be necessary in the development of a subset of gastric cancer.

In this study, it is interesting to note that cyclin D2 is not expressed in normal gastric tissues with unmethylated promoter. The reason for this discrepant finding between normal and cancer tissues may be related to the fact that cyclin D2 is a direct target of Myc in which its expression is further controlled by *β*-catenin (He *et al*, 1998; Bouchard *et al*, 1999). We and others have previously shown that aberrant *β*-catenin translocation can only be observed in gastric cancer tissues (To *et al*, 2001; Tong *et al*, 2001; Clements *et al*, 2002). Thus, it is reasonable to anticipate that cyclin D2 is absent in normal gastric tissues with intact Wnt signalling pathway.

Promoter methylation, if involved tumour-suppressor genes (Lee *et al*, 1997; Iida *et al*, 2000; Song *et al*, 2000; Leung *et al*, 2001), usually results in selective growth advantage that favours the survival of neoplastic cells. However, it is increasingly recognised that promoter hypermethylation can also be detected in genes other than tumour-suppressor genes. One example is the methylation of cyclooxygenase-2 (COX-2) in gastric and colorectal cancer (Toyota *et al*, 2000; Kikuchi *et al*, 2002). COX-2 is generally considered to promote cancer development, and suppression of COX-2 activity may have an antiproliferative effect on tumour. Several groups of investigators (Toyota *et al*, 2000; Kikuchi *et al*, 2002) indicated that the promoter of COX-2 gene is methylated in a proportion of gastric cancer but not in normal gastric tissues. Their findings raise the possibility that a subgroup of gastric or colorectal cancers may not be responsive to the growth inhibitory effect of COX-2 inhibitors. In this manner, cyclin D2 overexpression is associated with dysregulation of cell cycle and appears to promote tumorigenesis. Promoter hypermethylation of cyclin D2 with resultant loss of cyclin D2 may therefore have an antineoplastic effect on gastric cancer cells. However, it is important to recognise that cytosine methylation can also influence tumorigenicity by mechanisms other than gene silencing. Methylated cytosines can undergo spontaneous deamination resulting in C→T transitions and they are also preferred targets for G→T transversion mutations (Herman *et al*, 1996; Jones and Baylin, 2002). Thus, further studies are necessary to characterise the functional consequences of *cyclin D2* methylation in the process of gastric carcinogenesis.

In this study, methylation of *cyclin D2* promoter region appears to be a tumour-specific event since methylation was detected only in gastric cancer and cancer cell lines, but not in normal gastric mucosa. The detection of cyclin D2 mRNA expression in tumours with promoter hypermethylation may be related to the extreme sensitivity of the test, which can theoretically detect as little as 0.1% of methylated cells (Herman *et al*, 1996). Alternatively, a tumour may exhibit heterogeneity in *cyclin D2* methylation. In this regard, partially methylated promoter regions may reduce the level of transcriptional repression, resulting in partial loss of gene expression only (Hsieh, 1994). Notably, we showed that three cancer samples did not express cyclin D2 in the absence of methylation. This finding suggests that alternative pathways, such as homozygous deletion or genetic mutations, may account for loss of cyclin D2 in some tumours. Both of these events, however, have not been reported in gastric cancer.

*Cyclin D2* methylation was more frequent in older patients, suggesting that *cyclin D2* methylation may play a more important role in gastric carcinogenesis of elderly patients. However, we believe that methylation in *cyclin D2* is not an age-related phenomenon since methylation is not detected in any normal gastric mucosa including those tissues obtained from patients ⩾60 years. Moreover, previous studies in breast cancer and Burkitt's lymphoma suggest that methylation in *cyclin D2* is a tumour-specific event (Sinclair *et al*, 1995; Evron *et al*, 2001; Lehmann *et al*, 2002).

In summary, transcriptional silencing by promoter hypermethylation of the *cyclin D2* gene is detected in a subset of gastric cancer. Our results suggest that the development of a subset of gastric cancer is independent of cyclin D2 expression. Further study is necessary to determine the functional significance underlying the methylation-mediated transcriptional loss of cyclin D2 in gastric carcinogenesis.
